# Safety and efficacy of a generic fixed-dose combination of stavudine, lamivudine and nevirapine antiretroviral therapy between HIV-infected patients with baseline CD4 <50 versus CD4 ≥ 50 cells/mm^3^

**DOI:** 10.1186/1742-6405-4-6

**Published:** 2007-03-13

**Authors:** Weerawat Manosuthi, Sukanya Chimsuntorn, Sirirat Likanonsakul, Somnuek Sungkanuparph

**Affiliations:** 1 Bamrasnaradura Infectious Diseases Institute, Ministry of Public Health, Nonthaburi, 11000, Thailand; 2 Faculty of Medicine Ramathibodi Hospital, Mahidol University, Bangkok, 10400, Thailand

## Abstract

**Background:**

Antiretroviral therapy (ART) with a generic fixed-dose combination (FDC) of stavudine (d4T)/lamivudine (3TC)/nevirapine (NVP) is widely used in developing countries. The clinical data of this FDC among very advanced HIV-infected patients is limited.

**Methods:**

A retrospective cohort study was conducted among ART-naïve HIV-infected patients who were initiated a generic FDC of d4T/3TC/NVP between May 2004 and October 2005. Patients were categorized into 2 groups according to the baseline CD4 (group A: <50 cell/mm^3 ^and group B: ≥ 50 cell/mm^3^).

**Results:**

There were 204 patients with a mean ± SD age of 37.1 ± 8.9 years, 120 (58.8%) in group A and 84 (41.2%) in group B. Median (IQR) CD4 cell count was 6 (16–29) cells/mm^3 ^in group A and 139 (92–198) cells/mm^3 ^in group B. Intention-to-treat analysis at 48 weeks, 71.7% (86/120) of group A and 75.0% (63/84) of group B achieved plasma HIV RNA <50 copies/ml (*P *= 0.633). On-treatment analysis, 90.5% (87/96) in group A and 96.9% (63/65) in group B achieved plasma HIV RNA <50 copies/ml (*P *= 0.206). At 12, 24, 36 and 48 weeks of ART, mean CD4 were 98, 142, 176 and 201 cells/mm^3 ^in group A and 247, 301, 336 and 367 cells/mm^3 ^in group B, respectively. There were no differences of probabilities to achieve HIV RNA <50 copies/ml (*P *= 0.947) and CD4 increment at 48 weeks between the two groups (*P *= 0.870). Seven (9.6%) patients in group A and 4 (8.5%) patients in group B developed skin reactions grade II or III (*P *= 1.000). ALT at 12 weeks was not different from that at baseline in both groups (*P *> 0.05).

**Conclusion:**

Initiation of FDC of d4T/3TC/NVP in HIV-infected patients with CD4 <50 and ≥ 50 cells/mm^3 ^has no different outcomes in terms of safety and efficacy. FDC of d4T/3TC/NVP can be effectively used in advance HIV-infected patients with CD4 <50 cells/mm^3^.

## Background

Current antiretroviral treatment guidelines for HIV infection in adults and adolescents in the resource-limited settings recommend using two nucleoside reverse transcriptase inhibitors (NRTIs) plus one non-nucleoside reverse transcriptase inhibitor (NNRTI) as the first-line antiretroviral regimen [1,2]. Regimens based on these combinations are efficacious, are generally less expensive, have generic formulations and are available as generic fixed-dose combinations (FDC). To date, two NNRTIs are currently available for clinical use in the treatment of HIV disease. Either nevirapine (NVP) or efavirenz (EFV) has shown antiretroviral efficacy [3-5]. A recent large investigation has demonstrated that both drugs have similar antiviral efficacy among antiretroviral naïve HIV-infected patients [5].

Although EFV-based ART is an NNRTI-based antiretroviral therapy (ART) of choice according to the recommendation of recent antiretroviral treatment guidelines [1,2], NVP-based HAART has been extensively used in the developing countries due to its accessibility. Since 2002, the Thai Government Pharmaceutical Organization (GPO) has produced a FDC of 30 or 40 mg stavudine (d4T), 150 mg lamivudine (3TC) and 200 mg nevirapine (NVP). This combination formula makes dosing simple (one tablet twice daily) and facilitates drug supply procedure for the national ART program. The previous study of bio-equivalence showed that NVP concentrations were within international and manufacturer's standard [6]. In addition, World Health Organization (WHO) guideline recommends a combination of two nucleoside reverse transcriptase inhibitors (NRTIs) and one NNRTI as first-line regimen in resource-poor settings due to available evidences, clinical experience and programmatic feasibility for the wider introduction of ART [7]. The Medecins Sans Frontieres (MSF) cohort result recently demonstrated the efficacy and safety of generic FDC in preventing AIDS-related death in resource-limited settings [8].

There are some potential limitations of NVP-based ART including adverse drug reactions and low genetic barrier. Skin rash is the most frequently observed adverse event from NVP and manifests as a diffuse maculopapular rash or erythematous rash with or without constitutional symptoms. The risk of rash at any severity is greatest in the first six weeks [9]. However, the severe rashes have been reported [10-13].

To date, the data regarding safety and efficacy of the d4T/3TC/NVP FDC among advanced HIV-infected patients with very low CD4 is still limited. Patients in resource-limited settings usually present late with very low CD4 cell counts. We therefore conducted this retrospective cohort study to compare immunological and virological outcomes and adverse events of a generic FDC of d4T/3TC/NVP between HIV-infected patients who had baseline CD4 cell counts <50 cells/mm^3 ^and those who had CD4 cell counts ≥50 cells/mm^3^.

## Methods

A retrospective cohort study was conducted among ART-naïve HIV-infected patients who were initiated a generic FDC of d4T/3TC/NVP between May 2004 and October 2005 in Bamrasnaradura Infectious Diseases Institute, Ministry of Public Health, Nonthaburi, Thailand. The patients' identification numbers were identified from annual database of the institute. The data were extracted from the medical records. Inclusion criteria were as follows: (1) HIV-infected individuals ≥ 15 years old, (2) naïve to ART prior to FDC of d4T/3TC/NVP, (3) were initiated with a generic FDC of d4T/3TC/NVP, (4) used separate tablet of NVP 200-mg once-daily lead-in dose during the first 2 weeks, prior to escalation to 200 mg twice daily, (5) followed up at least two clinic visit. Exclusion criteria were as follows: (1) baseline serum creatinine level > 2.0 mg/ml, (2) baseline liver aminotransferase >3 times of upper normal limit, (3) currently active major opportunistic infections (OIs), and (4) receiving medications that have drug-drug interactions with NVP, including rifampicin and fluconazole.

All eligible patients were categorized into two groups according to their baseline CD4 cell counts: group A (CD4 cell count <50 cells/mm^3^) and group B (CD4 cell counts ≥ 50 cells/mm^3^). Factors including demographics, previous OIs, CD4 cell counts, plasma HIV RNA were studied and compared between the two groups. Patients were followed up for 48 weeks after initiation of a generic FDC of d4T/3TC/NVP. The parameters including CD4 cell counts, plasma HIV RNA and ALT were assessed at baseline, 12, 24, 36 and 48 weeks of ART. The primary outcome of interest was the proportion of patients who achieved plasma HIV RNA <50 copies/ml after 48 weeks of ART. The secondary outcomes were as follows: 1) the probability to achieve plasma HIV RNA <50 copies/ml, 2) the increment of the number of CD4 cell counts at 48 weeks of ART from baseline value and 3) the incidences of NVP-associated adverse reactions including skin rashes and hepatotoxicity that lead to drug discontinuation.

The severity of skin rashes was determined as Level I: erythema; Level II: diffuse maculopapular rash or urticaria; Level III: rash with constitutional symptoms, angioedema, serum sickness-like reactions, Stevens Johnson syndrome; Level IV: toxic epidermal necrolysis. The virological failure was defined as either a rebound plasma HIV RNA of >1,000 copies/ml after having previously undetectable value or lack of achievement to <50 copies/ml at 24 weeks of ART.

Mean (± SD), median (interquartile range, IQR) and frequencies (%) were used to describe patients' characteristics in both groups. *Chi*-square test and Mann-Whitney U test were used to compare categorical variables and continuous variables between the two groups, respectively. The proportion of patients with plasma HIV RNA <50 copies/ml after 48 weeks of ART were analyzed as intention-to-treat and on-treatment analysis. Missing data on plasma HIV RNA levels were taken to be greater than 50 copies/mL. The increment of CD4 cell counts between the two groups were compared by Mann-Whitney U test. The Kaplan-Meier test was used to estimate the probability of undetectable plasma HIV RNA at 12, 24, 36 and 48 weeks after ART and the median time to undetectable plasma HIV RNA. The patients were censored when they had virological failure or discontinued the FDC due to any causes. The patients who had been on drug holidays longer than 4 weeks were considered as lost to follow-up and censored at the date of first lost to follow-up visit. The log-rank test was used to compare the median time to undetectable plasma HIV RNA between the two groups. The multivariate Cox proportional hazard model was used to determine the chance of undetectable plasma HIV RNA after receiving treatment by adjusting for confounding factors, i.e. age, gender, previous OIs, baseline hemoglobin, CD4 cell counts (<50 versus ≥ 50 cells/mm^3^) and plasma HIV RNA at baseline. Statistical calculations were performed using SPSS program version 11.5 (SPSS Inc., Chicago, Illinois, U.S.A). A two-sided *P *value of less than 0.05 was considered statistically significant. The study was approved from the institute review board.

## Results

A total of 204 patients met entry criteria; mean (± SD) age was 37.1 ± 8.9 years and 60.3% were male. There were 120 (58.8%) patients in group A and 84 (41.2%) patients in group B. Table 1 shows baseline characteristics between the two groups. Median (IQR CD4 cell count was 6 (16–29) cells/mm^3 ^in group A and 139 (92–198) cells/mm^3 ^in group B. Group A had more previous opportunistic infections, higher baseline HIV RNA, ALP and ALT than group B (*P *< 0.05). Any causes of discontinuation of ART are shown in Table 2. There were no differences of the causes of discontinuation between the two groups. Of 204 patients, 162 patients had ALT values at 12 weeks after ART. Two of 95 (2.1%) patients in group A and 1 of 64 (1.6%) patients in group B had ALT elevation at grade 3–4 (*P *= 1.000).

**Table 1:** Baseline characteristics of 204 patients

**Characteristics**	**Group A (n = 120)**	**Group B (n = 84)**	***P *value**
Age, years, mean ± SD	37.6 ± 8.0	36.9 ± 10.1	0.289
Male gender	70 (58.3%)	53 (63.1%)	0.561
Body weight, Kgs, mean ± SD	54.3 ± 10.3	56.1 ± 13.8	0.766
Previous major OIs	41 (34.2%)	11 (13.1%)	0.001
Baseline hemoglobin, mg/dl, median (IQR)	10.7 (8.4–12.0)	11.5 (9.5–13.3)	0.035
Baseline CD4, cell/mm^3^, median(IQR)	6 (16–29)	139 (92–198)	<0.001
Baseline CD4%, median (IQR)	2 (1–2)	8 (0–13)	<0.001
Baseline plasma HIV RNA, copies/ml, median (IQR)	357,000 (187,000–727,750)	231,000 (69,750–645,250)	0.027
Baseline ALP, U/I, median (IQR)	91.0 (71.0–128.0)	74.5 (58.0–94.2)	<0.001
Baseline ALT, U/I, median (IQR)	32.0 (18.0–50.0)	21.5 (16.0–33.5)	0.003
Baseline total bilirubin, mg/dL, median (IQR)	0.5 (0.4–0.7)	0.5 (0.4–0.7)	0.532

**Table 2:** Causes of ART discontinuation between the 2 groups

**Causes of discontinuation**	**Group A (n = 120)**	**Group B (n = 84)**	***P *value**
NVP-associated skin rashes grade II, III and IV	11 (9.2%)	12 (14.3%)	0.269
Virological failure	9 (7.5%)	2 (2.4%)	0.129
Lost to follow-up	7 (5.8%)	5 (6.0%)	1.000
Died	4 (3.3%)	1 (1.2%)	0.651
Referred to other hospitals	1 (0.8%)	0*	1.000
d4T-associated lactic acidosis	1 (0.8%)	1 (1.2%)	1.000

* Substitute 0 as 1 to calculate *P *value

**Figure 1:**
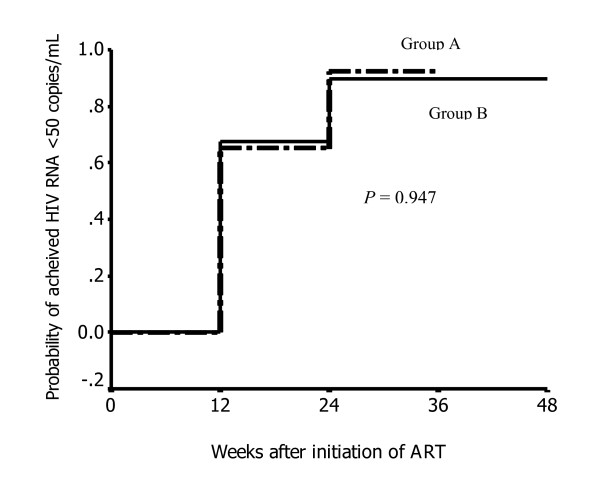
Probability of undetectable plasma HIV RNA between the 2 groups.

The results of the primary outcome are shown in Table 3. There were no differences of proportion of patients who achieved plasma HIV RNA <50 copies/ml between the two groups, either in intention-to-treat analysis or on-treatment analysis. Cox regression of possible risk factors for achieving undetectable plasma HIV RNA is shown in Table 4. The results of Kaplan-Meier analysis to estimate the probability of undetectable plasma HIV RNA after receiving treatment are shown in Figure 1. We found that such probabilities at 12-, 24- and 36-month were 65.1%, 92.4% and 92.4% for group A. The corresponding values were 67.6%, 89.7% and 89.7% for group B. There was no difference between the two groups (log rank test, *P *= 0.947). The Cox proportional hazard model including factors of age, gender, body weight, previous OIs, baseline hemoglobin, baseline CD4 cell counts and baseline plasma HIV RNA showed that patients in group A had similar chance of undetectable plasma HIV RNA with the patients in group B (HR = 0.986, 95%CI = 0.669–1.391, *P *= 0.934). The other factors were not associated with undetectable plasma HIV RNA after 48 weeks of ART (*P *> 0.05).

**Table 3:** Virological response between the two treatment groups at 48 weeks

	Percentage of patients who achieved HIV RNA< 50 copies/ml		Relative Risk, 95% confidence interval	*P *value
			
	Group A	Group B		
ITT*	71.7% (86/120)	75.0% (63/84)	0.843, 0.447–1.589	0.633
OT**	90.6% (87/96)	96.9% (63/65)	0.303, 0.063–1.453	0.202

* Intention-to-treat analysis ** On-treatment analysis

**Table 4:** Cox regression of possible risk factors for achieving undetectable plasma HIV RNA at 48 weeks

Risk factors	**HR**	**95% CI**	***P *value**
Age	1.000	0.980–1.020	0.993
Gender	0.948	0.661–1.513	0.774
Weight	0.998	0.983–1.013	0.778
Previous OIs	0.979	0.660–1.451	0.916
Hemoglobin	1.000	1.000–1.000	0.533
Baseline CD4 cell counts<50 cells/mm^3^	1.000	0.998–1.002	0.880
Baseline log plasma HIV-RNA	0.764	0.549–1.064	0.111

HR = Hazard ratio, 95% CI = 95% Confidence interval

**Figure 2:**
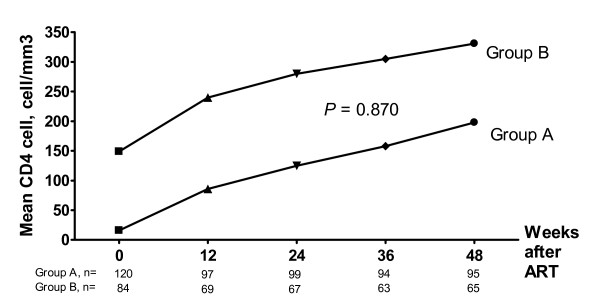
Immunological outcomes between the 2 groups at 48 weeks of ART.

Group A patients had a median CD4 cell count of 85, 125, 158 and 198 cells/mm^3 ^and group B patients had a median CD4 of 240, 280, 305 and 331 cells/mm^3 ^at 12, 24, 36 and 48 weeks, respectively (Figure 2). The increment of median (IQR) CD4 cell counts at 48 weeks from baseline values were not different between the two groups [179 (121–226) cells/mm^3 ^vs. 168 (99–273) cells/mm^3^, *P *= 0.870]. Eleven (9.2%) patients in group A and 12 (14.3%) patients in group B developed NVP-associated skin reactions grade II and III in which lead to the discontinuation of generic FDC of d4T/3TC/NVP (*P *= 1.000). Mean ± SD ALT when these 23 patients developed skin reaction was 31.5 ± 21.1 U/l. No patients in the present study developed skin reactions grade IV. By repeated measurement analysis, there were no differences of ALT at 12 weeks from baseline value in both groups (*P *> 0.05). No patients developed clinical hepatitis. Three (2.5%) patients in group A and 5 (6.0%) patients in group B developed stavudine-associated peripheral neuropathy (*P *= 0.278). Stavudine-associated symptomatic lactic acidosis was observed in 2 patients during the 48-week study period. Four patients in group A and one patient in group B died during the study period. The causes of death of these 5 patients were as follows: disseminated histoplasmosis 1, *E. coli *sepsis 1, MAC infection 1 and wasting syndromes 2.

## Discussion

The results from the present study demonstrate that HIV-infected patients who had baseline CD4 cell counts <50 cells/mm^3 ^had similar virological and immunological responses when compared to those who had baseline CD4 cell counts ≥ 50 cells/mm^3^. This finding can support the use of this generic FDC of d4T/3TC/NVP in very advanced HIV-infected patients in the resource-limited settings. According to the guideline for management of HIV-infected patients in Thailand, ART is recommended for all patients with history of AIDS-defining illness or asymptomatic patients with CD4 cells count less than 200 cell/mm^3^. Therefore, almost all patients in group B have baseline CD4 cell counts between 50 cells/mm^3 ^and 200 cells/mm^3^. As expected, group A patients have a higher proportion of previous opportunistic infections and a higher level of plasma HIV RNA.

The overall proportion of patients who achieved plasma HIV RNA <50 copies/ml was 73% (149 of 204) after 48 weeks of ART in an ITT analysis. Despite the fact that group A patients were severely immunocompromised (median baseline CD4 cell count of 6 cells/mm^3^), we found that 71% of patients achieved undetectable plasma HIV RNA after 48 weeks of ART. This number is comparable to the virological response in group B and similar to other studies that conducted in our country and in developed countries [5,14-16]. Moreover, this outcome is confirmed by the analysis of probability of achieving plasma HIV RNA <50 copies/ml as shown in Figure 1. Although the patient in group A had a significantly higher baseline plasma HIV RNA than that in group B, the rates of achieving undetectable plasma HIV RNA are not different between group A and group B, by either univariate or multivariate analysis.

The well-established predictors of long-term virological success include potency of ART regimen, adherence to treatment, low baseline viremia, higher baseline CD4 cell counts and rapid reduction of viremia in response to treatment [17,18]. In the present study, the difference baseline CD4 cell counts and baseline plasma HIV-RNA did not affect virological responses after 48 weeks of treatment. This may be explained by the fact that this study did not include patients with CD4 cell count of greater than 200 cells/mm^3^, as in previous studies. HIV-infected patients in developing countries usually present late with low CD4 cell counts as previously mentioned. A recent study in a developing country also demonstrated that there was a high rate of virological and immunological success after six months of HAART, irrespective of the pre-HAART viral load and CD4 cell count [19].

Overall, CD4 cell counts rise with immune recovery from receiving ART. The increment of CD4 cell counts after 48 weeks of ART is not blunted with very low baseline CD4 cell counts as shown in Figure 2. Even in subgroup of patient with baseline CD4 cell counts less than 10 cells/mm^3^, they can achieve effective immune recovery by given sufficient time after initiation of ART. Median (IQR) CD4 cell counts increase from 5 (3–7) cells/mm^3 ^to 151 (92–231) cells/mm^3 ^in this subgroup (data not shown). This response would be continued for many years into effective antiviral effect of ART.

Regarding discontinuation of ART, NVP-associated skin rashes is an important reason for discontinuation. In the present of NVP 200-mg once-daily lead-in for 2 weeks, NVP may cause a mild skin rash in 15 to 20% of patients; 5 to 10% of which discontinue treatment [5,20,21]. After 48 weeks of ART, 11.3% of patients in the present study developed skin rashes in which lead to permanent discontinuation of NVP. This rate is similar to that in the previous study in Thais [14,22]. Of note, Steven-Johnson's syndrome and toxic epidermal necrolysis were not observed in this cohort in whom almost all of patients (91%) in the study had baseline CD4 cell counts less than 200 cells/mm^3^. Furthermore, no factors (i.e., gender and CD4 cell counts) were associated with skin rashes in the present study according to the results of logistic regression analysis (data not shown). These findings suggest that there is no clinical factor to predict the occurrence of rash from NVP in patients with very low CD4 cell counts. However, this should be interpreted with caution due to the limitation of sample size. It would be beneficial if there are any factors that can predict this adverse event. Further study to determine immunologic and genetic factors that associated with rash is encouraged.

Although there are limited data regarding the impact on hepatotoxicity, we decided to exclude the patients who concurrently received rifampicin and fluconazole from the study. These drugs may potentially increase incidence of hepatotoxicity [23-25]. The previous studies demonstrated that woman with higher CD4 cell counts appear to be at a highest risk. A 12-fold higher incidence of symptomatic events was seen in woman with CD4 cell counts of >250 cells/mm^3 ^at the time of nevirapine initiation when compared with woman with CD4 cell counts of ≤ 250 cells/mm^3 ^(11.0% vs 0.9%). An increased risk was also seen in men with baseline CD4 cell counts >400 cells/mm^3 ^when compare with baseline CD4 cell counts ≤ 400 cells/mm^3 ^(6.3% vs 1.2%) [26-29]. The present study show that no patients developed clinical hepatitis after 48 weeks of follow-up. In addition, neither group A patients nor group B patients had significant increment of liver enzymes after 12 weeks of ART when compare to baseline values. Overall, the frequencies of clinical hepatitis and hepatic laboratory abnormalities were low in both groups. In the present cohort, stavudine-associated symptomatic lactic acidosis was observed in 2 patients after 48 weeks of treatment. Although this number is low, this well-established adverse event should be closely monitored in the further long-termed treatment. Currently, stavudine is not a first-line antiretroviral drug recommended in the recent guideline of developed country due to its significant toxicities [7]. Additionally, eight patients needed to discontinue stavudine due to peripheral neuropathy. Until more options are accessible, stavudine is still a part of a simplified strategy for scaling-up ART in resource-poor settings according to previously mentioned benefits.

The study has demonstrated the satisfactory clinical outcomes, the extent of immunological restoration and virological responses of a generic FDC of d4T/3TC/NVP in very advanced HIV-infected patients. These results provide the evidence of benefit from NVP-based ART in advanced HIV-infected patients. Thus, this may support the physicians to prescribe NVP-based ART regimen for these patients.

The present study has some limitations. The study design is a retrospective study, which is not the best study design to evaluate the efficacy of antiretroviral regimen. However, this study design is a comparative study that evaluated the efficacy NVP-based ART between the patients who had extremely low CD4 cell counts and those who had moderate level of CD4 cell counts. The results of the present study may provide useful clinical data for caring advanced HIV-infected patients in developing countries. In addition, some clinical data may be underestimated and some possible risk factors may not be included. Our study was based on a tertiary care center for HIV-infected patients. These study populations were cared by infectious diseases specialists and HIV-experienced medical team. Thus, the similar results might not be achieved in the general or community hospital in resource-limited setting. Liver enzymes were not performed during the first few weeks of ART. However, no patients developed clinical hepatitis during this period. Baseline hepatitis B and hepatitis C serology were not routinely performed prior to ART initiation. We did not have reference group of patients with CD4 cell counts greater than 200 cells/mm^3^. It would be more interesting if we can compare clinical outcomes between these groups. Finally, the sample size may be not large enough to detect small difference of efficacy and low incidence of adverse events, particularly hepatitis.

In conclusion, initiation of a FDC of d4T/3TC/NVP in HIV-infected patients with baseline CD4 cell count of <50 and ≥ 50 cells/mm^3 ^has no different outcomes in terms of safety and 48-week virological and immunological response. Generic FDC of d4T/3TC/NVP can be effectively used in advance HIV-infected patients with CD4 <50 cells/mm^3^.

## Abbreviations

HAART: Highly active anti-retroviral therapy, HIV: Human immunodeficiency virus, NRTIs: Nucleoside reverse transcriptase inhibitors, NVP: NNRTs: Non-nucleoside reverse transcriptase inhibitors, FDC: Fixed-dose combinations, Nevirapine, EFV: Efavirenz, d4T: Stavudine, 3TC: Lamivudine, ART: Antiretroviral therapy, OIs: Opportunistic infections, AST: Aspartate aminotransferase, ALT: Alanine aminotransferase

## Competing interests

The author(s) declare that they have no competing interests.

## Authors' contributions

WM participated in the design of the study and statistical analysis. SC participated in the design of the study. SL participated in the design of the study. SS participated in the design of the study and statistical analysis.
